# The impact of training on self-reported performance in reproductive, maternal, and newborn health service delivery among healthcare workers in Tanzania: a baseline- and endline-survey

**DOI:** 10.1186/s12978-022-01452-4

**Published:** 2022-06-20

**Authors:** Tumbwene Mwansisya, Columba Mbekenga, Kahabi Isangula, Loveluck Mwasha, Stewart Mbelwa, Mary Lyimo, Lucy Kisaka, Victor Mathias, Eunice Pallangyo, Grace Edwards, Michaela Mantel, Sisawo Konteh, Thomas Rutachunzibwa, Secilia Mrema, Hussein Kidanto, Marleen Temmerman

**Affiliations:** 1 School of Nursing and Midwifery, The Aga Khan University, Dar es Salaam, Tanzania; 2 School of Nursing and Midwifery, The Aga Khan University, Kampala, Uganda; 3 Centre of Excellence in Women and Child Health, The Aga Khan University, Nairobi, Kenya; 4 Aga Khan Health Services, Mwanza, Tanzania; 5 Regional Health Management, Mwanza, Tanzania; 6 Department of Obstetrics and Gynecology, Aga Khan University, Dar es Salaam, Tanzania

**Keywords:** Reproductive health, Intervention, Control, Self-reported performance, Training need analysis, Healthcare workers

## Abstract

**Background:**

Delivery of quality reproductive health services has been documented to depend on the availability of healthcare workers who are adequately supported with appropriate training. However, unmet training needs among healthcare workers in reproductive, maternal, and newborn health (RMNH) in low-income countries remain disproportionately high. This study investigated the effectiveness of training with onsite clinical mentorship towards self-reported performance in RMNH among healthcare workers in Mwanza Region, Tanzania.

**Methods:**

The study used a quasi-experimental design with pre-and post-intervention evaluation strategy. The baseline was compared with two endline groups: those with intervention (training and onsite mentorship) and those without. The differences among the three groups in the sociodemographic characteristics were analyzed by using chi-square test for categorical variables, independent-sample t-test for continuous variables and Mann–Whitney U test for ordinal or skewed continuous data. The independent sample t-test was used to determine the effect of the intervention by comparing the computed self-reported performance on RMNH services between the intervention and control groups. The paired-samples t-test was used to measure the differences between before and after intervention groups. Significance was set at a 95% confidence interval with p ≤ 0.05.

**Results:**

The study included a sample of 216 participants with before and after intervention groups comprising of 95 (44.0%) and 121 (56.0%) in the control group. The comparison between before and after intervention groups revealed a statistically significant difference (p ≤ 0.05) in all the dimensions of the self-reported performance scores. However, the comparison between intervention groups and controls indicated a statistical significant difference on intra-operative care (t = 3.10, df = 216, *p* = 0.002), leadership skills (t = 1.85, df = 216, *p* = 0.050), Comprehensive emergency obstetric and newborn care (CEMONC) (t = 34.35, df = 216, *p* ≤ 0.001), and overall self-reported performance in RMNH (t = 3.15, df = 216, *p* = 0.002).

**Conclusions:**

This study revealed that the training and onsite clinical mentorship to have significant positive changes in self-reported performance in a wide range of RMNH services especially on intra-operative care, leadership skills and CEMONC. However, further studies with rigorous designs are warranted to evaluate the long-term effect of such training programs on RMNH outcomes.

## Plain language summary

Reproductive maternal and newborn health (RMNH) in low- and middle-income countries continue to face critical challenges. Training healthcare workers especially using a combined approach (training followed by immediate clinical mentorship) in RMNH have been documented as an essential strategy to reduce maternal and neonatal mortality in low-and middle-income countries closer to those in high-income countries. This study investigated the effectiveness of a Continuous Professional Development (CPD) trainings on performance among healthcare workers in Mwanza Region. The study included a sample of 216 participants with before and after intervention groups comprising of 95 participants and control group comprising of 121 participants. The findings revealed that in comparison between before and after intervention groups all dimensions of the self-reported TNA questionnaire had a statistically significant difference. However, the comparison between intervention and controls groups indicated a statistical significant difference on leadership skills, intra-operative care, Comprehensive emergency obstetric and newborn care (CEMONC) and overall RMNH self-reported performance. In conclusion, the findings demonstrated that healthcare workers’ self-identified and prioritized training needs that are supported with clinical mentorship results in significant positive changes in performance across a wide range of RMNH tasks. Therefore, conducting TNA that is followed by training and mentorship according to the identified needs among healthcare workers plays a significant role in improving performance on RMNH services among healthcare workers.

## Introduction

Reproductive maternal and newborn health (RMNH) in low- and middle-income countries continue to face critical challenges. A large body of literature suggest that maternal mortality in low- and some middle-income countries is 50- to 100-fold higher than in high-income countries [1, 2]. In support, some literature indicate that maternal mortality ratio (MMR) is low in high-income countries at 1 per 5600 live births, whereas that in low-income and middle countries remains substantially high at 1 per 30 live births [3–6]. Relatedly, neonatal mortality in low- and middle-income countries is also around tenfold higher than in high-income countries [7]. Amidst these worrying statistics, skilled birth attendants have been documented as essential to reduce maternal and neonatal mortality in low-and middle-income countries to rates closer to those in high-income countries [8]. Clinical mentorship which is a system of practical training and consultation to foster continuous profession development has been recommended by WHO to be integrated with and immediately following initial training for yielding high-quality clinical care outcomes [9]. However, the effect of such a combined approach on RMNH healthcare workers’ performance remains unknown. This calls for evaluation of the impact of clinical mentorship as an integral component of the training among healthcare workers in RMNH services in low- and middle-income countries. Thus, this study, evaluated the effectiveness of CPD training (short course training followed by the mentorship program) on the self-reported performance in specific areas of RMNH service provision as identified using a Training needs analysis (TNA) tool.

Low- and middle-income countries continue to face critical shortages of human resource for health especially skilled birth attendants. Tanzania for example, is experiencing a notable shortage of skilled birth attendants [10]. The available data in Tanzania indicate an average shortage of 56% of skilled workforce with private sector being disproportionally affected [11]. Moreover, a systematic review has indicated that the absence of skilled obstetric providers to be associated with maternal mortality of 1% of all deliveries [12]. As a remedial strategy, the WHO has recommended the use of skilled birth attendants that are supplied with the necessary requirements including capacity building for provision of their services [13]. However, the unmet training needs for RNMH among healthcare workers in low- and middle-income countries remains disproportionately high. The persistently high skills gaps among healthcare workers continues to be cited as major factors associated with pregnancy outcomes and high maternal and antenatal mortality [2]. Nevertheless, Continuous professional development (CPD) programs have been reported to enhance healthcare workers’ knowledge and skills and improve the quality of healthcare, thereby contributing to reducing maternal and newborn mortality [14, 15]. Moreover, effective mentorship have been reported to contribute to the improvement of certain quality of health care outcomes [16]. Therefore, effective training programs with clinical mentorship might have potentials to improve the knowledge and skills of healthcare workers to meet needs in health service delivery.

Although CPD courses have been found to improve the competencies of healthcare workers [17–19]. Tanzania currently has no documented formal program that responds to the training needs that are identified by healthcare workers themselves. Likewise, few of the training programs conducted to address healthcare workers skills gaps were delivered without inclusion of mentorship component to synergize the knowledge and skills gained by healthcare workers. Unfortunately, adopting and implementing international standards and guidelines in some low- and middle-income settings such as Tanzania may be difficult because of limited human resources, limited funding, cultural factors, irrelevance, limited attainability, and geographic challenges [20]. The training that is complemented by mentorship program has been found to be effective in improving healthcare workers skills [16, 21].

It is within this background, the present study aimed at investigating the effectiveness of tailored CPD trainings for healthcare workers on the self-reported performance in RMNH care in Mwanza Region. The findings derived from a large-scale project on RMNH which included a training component followed by clinical mentorship of healthcare workers. The details of the project on Improving Access to Reproductive, Maternal and Newborn Health in Mwanza, Tanzania (IMPACT) have been described elsewhere [22]. Briefly, as part of IMPACT project, participants’ training needs were evaluated before commencement of interventions by using the Training needs analysis (TNA) questionnaire. Then, local and international RMNH guidelines were examined, and contents adapted to develop training packages and subsequent clinical mentorship sessions. The training program focused on the gaps identified during training needs assessment, including basic and comprehensive obstetric and neonatal care, family planning, respectful maternity care, gender sensitivity, and use of local research evidence. We hypothesized that the training program would lead to increased self-reported performance in relation to the targeted training areas among healthcare workers. The findings of this study offer insights on the design of effective trainings that is supplemented by the onsite clinical mentorship for improving RMNH service delivery in low resource settings.

## Materials and methods

### Setting

Details of the study setting have been reported elsewhere [22, 23]. Briefly, the study was conducted in Mwanza Region, which is located in the northern part of Tanzania. The region is part of the Lake Zone, where the MMR was 453 deaths per 100,000 live births and the under-five mortality ratio was 88 deaths per 1000 live births in the 10-year period preceding the 2015/16 Tanzania Demographic and Health Survey [11]. Mwanza Region is one of five regions in Tanzania prioritized by the Tanzanian Government because of its poor RMNH indicators [11]. Understanding the training needs of healthcare workers in Mwanza Region formed an important entry point for IMPACT project in seeking to increase their contribution toward improving RMNH indicators.

### Study population

All healthcare workers responsible for RMNH service provision that were present in the selected facilities at the time of the survey were eligible to participate. The specific inclusion and exclusion criteria were as follows.

### Inclusion criteria

1. All healthcare workers aged 18 years and above.
2. Healthcare workers who could understand and communicate in the Kiswahili or English languages.
3. Healthcare workers who were working in RMNH.
4. Healthcare workers who were willing to provide informed consent and participate in this study.

### Exclusion criteria

1. Healthcare workers who were found in RMNH but who did not usually work in such a unit.
2. Healthcare workers that were unable to answer questions because of physical or mental impediments.
3. Healthcare workers that were not willing to participate.

### Study design

**Fig. 1:**
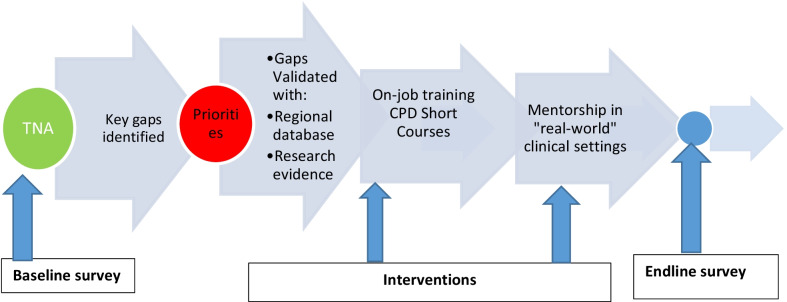
Study design flow chart

This was a quasi-experimental design with pre-and post-test evaluation strategy. This design has been widely used in healthcare settings previously [17, 24, 25]. Tailored training based on the identified and prioritized needs was implemented through a series of short courses. These courses were followed by a mentorship program that aimed at synergizing and complementing the knowledge and skills gained through the training courses. A similar approach was used in the mentorship program where mentees identified gaps in clinical skills that would guide the agenda for each mentorship visit. These courses were delivered at 36 health facilities in Mwanza Region. Three years after the training and a year mentorship program, the same health facilities were sampled and the healthcare workers in RMNH in these health facilities were recruited for a post-test survey. Figure 1 presents the flow of the study.

### Intervention (training program)

The training needs analysis was conducted at baseline to identify areas where capacity building was needed. After the baseline survey, the identified training needs were prioritized based on participants’ responses and validated with regional database and current evidence in literature. Short courses to address these areas were developed, some of which were adapted from national or international guidelines, as noted above. The topics covered by the short courses are outlined in Table 1 below. The number of trainees were determined by the identified Training needs gap, priority of each course and available resources. The course delivery included a combination of face-to-face sessions, simulation, and practical sessions in clinical environment under supervision of accredited trainers. The trainees were drawn from the participating health facilities in Mwanza Region and received more than one short course. Moreover, the short courses were followed by clinical mentorship in real-life clinical settings. The mentorships design involved experienced medical specialists and nurses/midwives with experience of over 15 years of serving as master mentors to local mentors (nurses & midwives and clinicians) in RMNH services. The Master mentors conducted a monthly mentorship visit to support trained healthcare providers in their healthcare facilities. A public health specialist (KI) with over 10 years of clinical experience in the same setting provided technical oversight and coordination of the mentorship intervention. Local mentors were identified from a list of trained healthcare workers based on their competencies, commitment, and availability for the mentorship program. The mentors were trained and standardized to a relatively equal capacity for training reproductive healthcare workers. The combine approach (training and onsite mentorship) has been implemented by several other studies and found to improve performance in clinical settings [19, 26–28].

**Table 1:** List of short courses conducted by the IMPACT project in Mwanza region

	Short courses	Number of trainees
1	Infection prevention and control	80
2	Standards-Based Management and Recognition	80
3	Health Management Information System	90
4	Referral	80
5	Health system management	114
6	Basic emergency obstetric and newborn care	104
7	Trainer of Trainers or comprehensive emergency obstetric and newborn care	30
8	Adolescent Sexual Reproduction Health	80
9	Respectful maternity care and gender responsive RMNH	80
10	Post abortion care	80
11	Focused antenatal care	80
12	Comprehensive emergency obstetric and newborn care	80
13	Family planning	80
14	Research methods for clinicians	40
15	Postnatal care	80
16	Community healthcare workers training and supervision	80
17	Clinical mentorship program	150

### Data collection tool

A training needs analysis (TNA) questionnaire was developed for RMNH providers at the primary (dispensary and health center) and secondary (health center and district and designated district hospital) levels. The tool was adapted from the Hennessy-Hicks TNA instrument [29, 30], which has been psychometrically tested for reliability and validity and adopted by the WHO [29]. Several other TNA questionnaires were considered, including the Professional Nurse Self-Assessment Scale of Clinical Core Competencies [31], Addiction Medicine Training Need Assessment Scale [32], and Community Competence-based Questionnaire [33]. However, the Hennessy-Hicks TNA questionnaire was selected because it was specifically developed to evaluate training requirements of healthcare professionals and facilitate the subsequent use of the findings for prioritizing and meeting local training needs [29]. The adapted 49 items TNA questionnaire measures a wide range of skills and have been validated in Kiswahili [23] the most spoken language in Tanzania. The related individual items of TNA questionnaire were grouped to make eight dimensions (intra-operative care, research skills, leadership skills, general reproductive health skills, CEMONC, family planning, gender in RMNH and total performance in RMNH). The dimension comprised of self-reported scores computed from the constituent items. The general RMNH skills dimension included the following items: providing client/patient friendly reproductive, maternal, child, and adolescent health services, understanding and using maternal, newborn and child health (MNCH) score cards (WHO), providing focused antenatal care (FANC) according to WHO guidelines, offering malaria diagnosis with rapid diagnostic testing (RDT), providing malaria treatment in pregnancy, providing malaria treatment in pregnancy, providing education and counselling around voluntary counselling and testing (VCT) for HIV/AIDS, providing education, counselling and support around HIV/AIDS prevention, care, and management for adolescents, competently managing uncomplicated deliveries, planning and organizing an individual patient’s care, evaluating patients’ psychological and social needs, implementing effective infection control strategies, and implementing effective disease surveillance and reporting. Leadership dimension included organizing your own time effectively, personally coping with change in the health service delivery, working as a member of a team, assuming a leadership role, developing leadership skills, mentoring and guiding other staff, and training, supervising and managing community health workers. The items for intra-operative care were providing care and education for cervical cancer screening and treatment, feeling confident in providing surgical and anesthetic care. Family planning items were providing family planning services to women and men in a union, family planning services to unmarried/single women and men, and providing information, education, counselling, or family planning services to adolescents. Gender in RMNH items included understanding gender equality issues related to RMNH, delivering gender sensitive RMNH services, identifying cases of sexual- and gender-based violence and knowing how to counsel and make appropriate referrals. Comprehensive emergency obstetric and newborn care (CEMONC) items were utilizing the partograph for every woman in labor, providing basic emergency obstetric and newborn care (BEmONC), managing severe intra- and postpartum hemorrhage, responding effectively to women suffering from severe pre-eclampsia and eclampsia, effectively resuscitating newborns using the newborn bag and mask, identifying dangerous signs and complications in childbirth and effectively managing maternal and newborn referral for further investigations or treatment, providing education and counselling on prevention of mother to child transmission of HIV, providing education, counselling and support to mothers in early initiation of breastfeeding and exclusive breastfeeding for 6 months, implementing the maternal infant and young child nutrition (MIYCN) program, Offering the Tanzania expanded program for immunization, understanding vaccine management and logistics, and proficient on injection safety and infectious waste management. Research audit activities items included undertaking effective data reporting and monitoring of service delivery, statistically analyzing your own data and using health facility data to understand local health challenges and inform service delivery, identifying research needs and designing locally relevant research, accessing research resources and influencing evidence-based services provision. The TNA questionnaire requires the participants to rate on: (a) how important is the task to their caring role (Rating A) and (b) how well the task is currently performed (Rating B) [20], and the difference in scores indicates the training needs among the studied tasks [20]. Moreover, the responses to the TNAQ are on a seven-point Likert scale: “not well” (1), “slightly well” (2), “quite well” (3), “moderately well” (4), “well” (5), “very well” (6), and “extremely well” (7). The higher the score the higher the self-rated capability to perform the task.

### Data collection procedure

Baseline data collection took place in August 2017 and endline data were collected from September to October, 2020. The team of two researchers and four research assistants collected the data. During data collection, the person in-charge of the selected facility identified RMNH personnel. The participants were those healthcare workers providing RMNH services and present at the time of the survey. The questionnaires were only given to these selected participants for completion. Participants were requested to assess their own performance on specific RMNH services/activities through a self-administered, confidential, paper-based questionnaire. Each questionnaire item was rated on a seven-point Likert scale. Participants were also asked to identify areas in which they most wanted to receive additional training and note the trainings that they had most recently completed. Research assistants were available to answer questions and clarify elements of the questionnaire as needed. Returned questionnaires were checked for completeness and accuracy before the research team left each health facility.

### Data analysis

SPSS version 25.0 was used for data entry and statistical analyses. Data from the questionnaires were reviewed to identify consistencies and differences, and then coded and quantified. The data were then manually entered into a password-protected database via an entry screen that performed validation checks for accuracy. Participants with significant missing data were excluded from analysis. We evaluated the normality of the TNA questionnaire score distribution using the Shapiro–Wilk test for self-reported performance at baseline and end line. The Shapiro–Wilk test confirmed the variable scores followed a normal distribution (*p* > 0.05).

The group differences in the sociodemographic characteristics were analyzed by using chi-square test for categorical variables and t-tests for continuous variables. The Mann–Whitney U test was used to test the median differences or skewed sociodemographic data. The individuals scores for general RMNH, leadership, intra-operative care, family planning, gender in RMNH, CEMONC and research audit activities were computed from the cluster of TNA items for both paired groups (before and after intervention groups) and the control group.

In the current study, the independent samples t-test was used to analyze the group differences in the self-reported performance scores between the intervention (those with training and mentorship) and controls (those without training and mentorship). The independent sample t-test was used to determine the effect of the intervention by comparing the computed self-reported participant’s performance scores on general RMNH, leadership, intra-operative care, family planning, gender in RMNH, CEMONC, research audit activities and overall performance between the intervention and control groups. To measure the long-term effect of the intervention, the paired-samples t-test was used for comparing the mean differences between the before and after intervention groups. Significance was set at a 95% confidence interval with p < 0.05.

### Ethical considerations

Ethical Approval to conduct this study was obtained from the National Institute for Medical Research in Tanzania (Registration Certificate: NIMR/HQR/R.8a/Vol.IX/2517) after review by the Aga Khan University Institutional Review Board in Tanzania. Permission to conduct this study obtained from the Mwanza Regional Administrative Secretary. All participants provided oral and written informed consent after receiving an explanation of the benefits, potential harm, duration of the questionnaire, and their right to refuse or withdraw from the study at any point.

## Results

### Participants

The study included a sample of 216 participants with the intervention group comprising of 95 (44.0%) and the control group 121 (56.0%) participants. Among 95 participants who received training and mentorship, 52 (54.7%) had received at least one of the IMPACT project training courses, 26 (27.4%) received two courses, 15 (15.8%) received three courses and two (2.1%) received four courses. The independent t-test was used to test the mean difference in age, Mann–Whitney test was used to determine the mean difference in duration of employment and in RMNH services and chi-square test was used to determine the statistical difference in sex between intervention and controls groups. As indicate in Table 2, participants differed (*p* ≤ 0.05) on their age and duration of employment in RMNH.

**Table 2:** Comparison of participants’ sociodemographic characteristics between the intervention and control groups

	Intervention group (n = 95)		Control group (n = 121)		Statistical tests		
	Mean	Sd	Mean	Sd	t/χ^2^	df	p-value
Age	38.31	9.13	35.75	9.19	2.02		0.045
Duration in employment	11.23	9.02	8.90	8.34	–	–	0.023
Duration in RMNH	8.61	8.93	7.52	7.93	–	–	0.331
Sex							
Male	29 (35%)		30 (35%)		0.88	1	0.348
Female	66 (23%)		91 (32%)				

*RMNH* reproductive, maternal, and newborn health

### Health facility characteristics

Comparison of the health facility characteristics showed that the facilities were comparable in terms of type and the kind of support they received (government or private/faith based) (Table 3).

**Table 3:** Comparison of health facility information

Variable	Endline (n = 216)		Statistical tests		
	Intervention group (n = 95)	Control group (n = 121)	χ^2^	df	p-value
Level of health facility					
Dispensary	31(37%)	23 (28%)	5.27	4	0.072
Health center	30 (23%)	46 (36%)			
Hospital	34 (22%)	52 (33%)			
Type of support					
Government	82 (25%)	104 (32%)	0.01	1	0.939
Faith-based	13 (28%)	17 (37%)			

### Paired-sample t-test on self-reported performance scores in the intervention group

Comparisons on self-reported performance scores between the before and after intervention groups indicated that the computed self-reported scores on general RMNH, leadership, intra-operative care, family planning, gender in RMNH, CEMON and research audit activities and overall performance to be higher in the after intervention group as compared to the before intervention group and with a statistical significant differences (*p* ≤ 0.05). The details are provided in Table 4.

**Table 4:** The paired sample t-test on pre-and post-training self-reported performance scores in the intervention group

Variable	Groups	N	Mean	Std. Dev.	Mean difference	t	df	p-value
Intra-operative care	Before	95	7.61	3.29	7.93	14.96	94	≤ 0.001
	After	95	16.32	4.89				
Research skills	Before	95	21.72	3.74	1.75	3.57	94	≤ 0.001
	After	95	23.47	6.02				
Leadership skills	Before	95	40.88	8.54	6.69	5.33	94	≤ 0.001
	After	95	47.57	9.30				
General RMNH skills	Before	95	69.29	52.70	11.29	0.29	94	0.044
	After	95	80.58	7.82				
CEMONC	Before	95	64.25	14.36	14.44	8.29	94	≤ 0.001
	After	95	78.69	9.18				
Family planning	Before	95	14.49	16.91	2.42	4.80	94	≤ 0.001
	After	95	16.91	3.29				
Gender in RMNH	Before	95	12.75	7.31	8.97	10.82	94	≤ 0.001
	After	95	21.72	3.74				
Total performance in RMNH	Before	95	238.97	64.90	44.39	5.89	94	≤ 0.001
	After	95	283.35	34.02				

*CEMONC* comprehensive emergency obstetric and newborn care, *RMNH* reproductive, maternal, and newborn health

### Independent-sample t-test between the intervention and control groups on the self-reported scores

To test whether the difference in means between the intervention and control was statistically significant, the independent-samples t-test was performed. The results of the t-test revealed that there was a statistically significant difference in self-reported performance on intra-operative care (t = 3.10, df = 216, p = 0.002), leadership skills (t = 1.85, df = 216, p = 0.050), CEMONC (t = 34.35, df = 216, p ≤ 0.001), overall self-reported performance in RMNH (t = 3.15, df = 216, p = 0.002). However, there was no statistically significant difference between the intervention and control groups on research skills, general reproductive health skills, family planning, and gender in reproductive health. Table 5 shows the details for mean differences between the intervention and control groups.

**Table 5:** Independent-sample t-test on the pre- and post-training self-reported performance scores between the intervention and the control groups

Variable	Groups	N	Mean	Std. Dev.	Mean difference	*t*	df	*p*-value
Intra-operative care	Intervention	95	16.31	3.19	1.52	3.10	216	0.002
	Control	121	14.80	4.01				
Research skills	Intervention	95	26.52	5.83	2.33	1.85	216	0.059
	Control	121	24.18	10.79				
Leadership skills	Intervention	95	47.58	9.30	2.15	1.76	216	0.050
	Control	121	45.43	8.01				
General RMNH skills	Intervention	95	76.71	10.10	2.68	1.74	216	0.075
	Control	121	74.02	11.24				
CEMONC	Intervention	95	78.51	9.16	6.05	4.35	216	≤ 0.001
	Control	121	72.45	10.84				
Family planning	Intervention	95	16.91	3.29	0.72	1.03	216	0.263
	Control	121	16.19	5.49				
Gender in RMNH	Intervention	95	21.44	3.70	0.87	1.52	216	0.122
	Control	121	20.57	4.39				
Total performance in RMNH	Intervention	95	283.96	33.96	16.32	3.15	216	0.002
	Control	121	267.64	39.42				

*CEMONC* comprehensive emergency obstetric and newborn care, *RMNH* reproductive, maternal, and newborn health

## Discussion

This study aimed to evaluate the effectiveness of CPD training on self-reported performance in specific areas of RMNH service provision as identified using a TNA tool. The purpose was to offer insights that could inform the design of effective trainings for improving RMNH service delivery in low resource settings.

This study demonstrated an overall statistically significant positive changes among RMNH healthcare workers following the intervention (training and mentoring). Comparing to the control group, the intervention group was found with significant improvement on self-reported performance in provision of overall RMNH services, leadership skills, CEMONC and on intra-operative care, indicating the impact of training and onsite mentorship intervention. However, some dimensions including research skills, general RMNH skills, family planning, and gender in RMNH were found to be not statistically significant. The possible explanation for such findings might be related to that after the initial assessment the intervention group might have gained the comparable level of performance to the control group in research skills, general RMNH skills, family planning, and gender sensitivity. These findings mirror the current scholarly discussion on RMNH. Research evidence continue to show that healthcare providers improved their performance in obstetric care, including conducting surgical procedures, when they received the correct training and support [34–36].

Low- and middle-income countries, particularly Sub-Saharan African countries, are facing a crisis in available human resources for health, including insufficient providers of RMNH services. The literature suggests that evidence-based deployment and training policies have potentials to improve service provision in low-resource settings, including healthcare settings in rural Africa [37]. Therefore, the combined training and mentorship approach on a wide range of RMNH skills implemented in this study has been confirmed to be effective for improving self-reported performance in RMNH training.

This findings indicates that the overall self-reported performance on RMNH was significantly higher among the intervention group compared to control group following the post-test scores. This indicates that the training and onsite mentorship program enabled the healthcare workers to develop a wide range of skills in RMNH that are necessary in their service provision. Similar to this study findings, a previous study indicated that a combined exposure to certain procedures in reproductive health has a cumulative positive impact on intention to provide such services in the future [38]. Specifically, the training that is complemented by mentorship program has been found to be effective in improving healthcare workers skills [16, 21]. Therefore, this study findings implies that short courses training that are supplemented by the onsite mentorship might enable healthcare workers to successfully perform the RMNH services.

The study revealed a significant positive change in self-reported performance pre-post-test scores in leadership skills in intervention group as compared to control group, including mentoring, guiding other staff, supervising and management of community health workers. Good leadership skills are essential for optimal RMNH team performance [39, 40]. In this study, health system management and leadership skills were integrated in each of the RMNH training courses. These findings are similar to those reported in previous studies. For instance, Block, Dehlendorf [39] reported that additional procedural training accompanied by mentorship of leadership skills was the most critical component of a program on successful provision of reproductive health services. Studies further indicate that Leadership competencies such as demonstrating appreciation for team members and creating connections between colleagues have been found to improve morale and increase retention, innovation, and productivity, and also have potential to prevent burnout [41, 42]. Another study from India suggested that transformative learning including interdisciplinary leadership competencies such as self-awareness, vision, self-regulation, motivation, decisiveness, integrity, interpersonal communication skills, strategic planning, team building, innovation, and being an effective change agent are effective for an interdisciplinary team of healthcare providers [43]. The present study provided the evidence on the impact of training on general health system management, further studies may be needed to explore the specific leadership skills required for RMNH in low-income settings.

A comparison of the post-test scores between the intervention and control groups indicated that the intra-operative care and CEMONC scores increased significantly for health care workers in the intervention group compared to the control group. This findings provides evidence that the training and onsite mentorship intervention to have a statistically significant positive impact on the RMNH service performance among healthcare workers. The finding of the current study is consistent with another study from the same context that used a multimodal mentorship intervention for improving the quality surgical care [44]. Moreover, other studies reported that a mentorship intervention that follows training, uses a side-by-side approach, high-quality mentee–mentor relationships, and provides nonjudgmental feedback leads to success in low-resource settings [28, 44, 45]. Therefore, it is plausible that the training that supplemented with mentorship might be the effective approach for skills improvement among healthcare workers.

Some dimensions of TNA including research skills, general reproductive RMNH skills, family planning and gender in RMNH, though might be important in RMNH, in the current study were found with no statistical significant difference between the intervention and the control group. The reasons for such findings might be related to the government policies and attitude of healthcare workers towards family planning, research, gender and some general issues related to RMNH. For instance, the research capacity of health personnel, financial resources, positive reward systems, and collaborative relationships have been reported to be fundamental for improving RMNH services [46]. However, in low- and middle-income countries such as Tanzania, most available funds, expertise, and other resources come from the North–South collaboration, and little attention is directed to research activities [47]. In the present study, the comparison between intervention and control groups yielded no significant changes between the intervention and control groups in self-reported research skills performance. Therefore, strengthening research capacity in low- and middle-income countries has been reported to be a powerful, cost effective, and sustainable way of advancing health, healthcare, and development [48]. Strengthening research capacity may enable healthcare providers to undertake research based on their local needs and priorities that can advance RMNH services and related leadership skills in low- and middle-income countries. Innovative interventions for changing attitudes and building research capacity in research management, proposal writing for grants, and report writing may provide positive changes in performance in research.

The present study provides evidence of positive changes among healthcare workers in terms of the self-reported performance in identified and prioritized areas in overall performance of RMNH services, leadership, CEMONC and intra-operative care. This intervention (training program) was unique because it covered the range of knowledge and skills in RMNH and was supplemented by mentorship program in the real-life clinical setting. Mentorship interventions have been reported to have a statistically significant positive impact on competencies among healthcare providers [49]. The CPD courses and mentorship program were effective and may be less expensive than other training options because they used local trainers and mentors. However, cost analysis may be needed to substantiate the cost of the employed approach.

The current study have some strength worth mentioning. This study used a combined approach of job and onsite clinical mentorship. Studies have indicated that many trainees lose their skills soon after training, therefore the combined approach used in this study ensures retention of knowledge and skills. Moreover, there is a need for continuous use of low dose high frequency training model [18]. This study is without limitations. At the time the endline survey was conducted, there were varying durations on the CPD courses (6 months to 3 years). Therefore, the observed differences between baseline and endline scores may partly result from changes in the learning climate (e.g., training from other partners) and accumulation of experience through clinical practice over this period. However, having the control to the intervention group at the endline provided an opportunity for the control of such biases. Second, the baseline was conducted through the TNA questionnaire that may have highlighted existing training needs in an individual participant than the work related self-reported performance. Therefore, the healthcare workers may have focused on improving their performance on the identified gaps. Third, though the health facilities were selected randomly, participants were recruited using convenience sampling. This means the findings may not be generalizable to all healthcare providers in Tanzania.

## Conclusions

In conclusion, the present study was conducted in a low-income setting using a TNA tool to identify, prioritize, implement, and evaluate the effectiveness of trainings that are supplemented by the mentorship program on self-reported performance in aspects of RMNH services among healthcare workers. The findings revealed that training based on healthcare workers’ identified and prioritized needs according to their healthcare facilities and supported with clinical mentorship resulted in significant positive changes in self-reported performance across a wide range of RMNH services. Therefore, conducting TNA that is followed by training and mentorship according to the identified needs among healthcare workers plays a significant role in improving their performance. However, further studies with rigorous designs are warranted to evaluate the long-term effect of such a training program on pregnancy and newborn outcomes.

## Data Availability

The data that support the findings of this study are available from the Aga Khan University Monitoring and Evaluation Research Unit (MERL). There are some restrictions to the availability of these data due to license, so the data are not publicly available. However, the data may be made available from the authors upon reasonable request and with permission of the Aga Khan University MERL.

## Abbreviations

BEmONC: Basic emergency obstetric and newborn care
CEmONC: Comprehensive emergency obstetric and newborn care
CPD: Continuous professional development
IMPACT: Improving access to reproductive, maternal and newborn health in Tanzania
TNAQ: Training Need Analysis Questionnaire
RMNH: Reproductive, maternal, newborn, health
MMR: Maternal mortality rate
NIMR: National Institute for Medical Research
TDHS: Tanzania demographic health survey
MOHCDGEC: Ministry of Health, Community Development, Gender, Elderly and Children
WHO: World health organization
URT: United Republic of Tanzania

## References

[1] Alkema L, Chou D, Hogan D, Zhang S, Moller AB, Gemmill A (2016). Global, regional, and national levels and trends in maternal mortality between 1990 and 2015, with scenario-based projections to 2030: a systematic analysis by the UN maternal mortality estimation inter-agency group. Lancet.

[2] Goldenberg RL, McClure EM, Saleem S (2018). Improving pregnancy outcomes in low- and middle-income countries. Reprod Health.

[3] Semali IA, Tengia-Kessy A, Mmbaga EJ, Leyna G (2015). Prevalence and determinants of stunting in under-five children in central Tanzania: remaining threats to achieving millennium development goal 4. BMC Public Health.

[4] Clark J (2015). Child deaths plummet but many countries miss millennium development goal. BMJ.

[5] Vogel JP, Pileggi-Castro C, Chandra-Mouli V, Pileggi VN, Souza JP, Chou D (2015). Millennium development goal 5 and adolescents: looking back, moving forward. Arch Dis Child.

[6] Fernandes BB, Nunes FB, Prudencio PS, Mamede FV (2015). Epidemiological research of the maternal deaths and compliance with the fifth millennium development goal. Rev Gaucha Enferm.

[7] Mariani G, Kasznia-Brown J, Paez D, Mikhail MN, Salama DH, Bhatla N (2017). Improving women’s health in low-income and middle-income countries. Part I: challenges and priorities. Nucl Med Commun.

[8] Wu H, Zhao M, Liang Y, Liu F, Xi B (2021). Maternal age at birth and neonatal mortality: associations from 67 low-income and middle-income countries. Paediatr Perinat Epidemiol.

[9] WHO (2005). WHO recommendations for clinical mentoring to support scale-up of HIV care, antiretroviral therapy and prevention in resource-constrained settings.

[10] Shimamoto K, Gipson JD (2015). The relationship of women’s status and empowerment with skilled birth attendant use in Senegal and Tanzania. BMC Pregnancy Childbirth.

[11] TDHS. Tanzania demographic health survey (TDHS) 2010 and 2015/16. 2016.

[12] Vieira C, Portela A, Miller T, Coast E, Leone T, Marston C (2012). Increasing the use of skilled health personnel where traditional birth attendants were providers of childbirth care: a systematic review. PLoS ONE.

[13] Walker T, Woldegiorgis M, Bhowmik J (2021). Utilisation of skilled birth attendant in low- and middle-income countries: trajectories and key sociodemographic factors. Int J Environ Res Public Health.

[14] Frenk J, Chen L, Bhutta ZA, Cohen J, Crisp N, Evans T (2010). Health professionals for a new century: transforming education to strengthen health systems in an interdependent world. Lancet.

[15] Ona S, Easter SR, Prabhu M, Wilkie G, Tuomala RE, Riley LE (2019). Diagnostic validity of the proposed Eunice Kennedy Shriver national institute of child health and human development criteria for intrauterine inflammation or infection. Obstet Gynecol.

[16] Schwerdtle P, Morphet J, Hall H (2017). A scoping review of mentorship of health personnel to improve the quality of health care in low and middle-income countries. Glob Health.

[17] Alnazly EK (2018). The impact of an educational intervention in caregiving outcomes in Jordanian caregivers of patients receiving hemodialysis: a single group pre-and-post test. Int J Nurs Sci.

[18] Cavicchiolo ME, Cavallin F, Bertuola F, Pizzol D, Segafredo G, Wingi OM (2018). Effect of a low-dose/high-frequency training on real-life neonatal resuscitation in a low-resource setting. Neonatology.

[19] Lutenbacher M, Elkins T, Dietrich MS, Riggs A (2018). The efficacy of using peer mentors to improve maternal and infant health outcomes in hispanic families: findings from a randomized clinical trial. Matern Child Health J.

[20] Maaloe N, Meguid T, Housseine N, Tersbol BP, Nielsen KK, Bygbjerg IC (2019). Local adaption of intrapartum clinical guidelines, United Republic of Tanzania. Bull World Health Organ.

[21] Jayanna K, Bradley J, Mony P, Cunningham T, Washington M, Bhat S (2016). Effectiveness of onsite nurse mentoring in improving quality of institutional births in the primary health centres of high priority districts of Karnataka, South India: a cluster randomized trial. PLoS ONE.

[22] Orwa J, Mantel M, Mugerwa M, Brownie S, Pallangyo ES, Mwasha L (2019). Maternal healthcare services use in Mwanza Region, Tanzania: a cross-sectional baseline survey. BMC Pregnancy Childbirth.

[23] Mwansisya T, Mbekenga C, Isangula K, Mwasha L, Pallangyo E, Edwards G (2021). Translation and validation of training needs analysis questionnaire among reproductive, maternal and newborn health workers in Tanzania. BMC Health Serv Res.

[24] Agrawal N, Bhargava S, Usmanova G, Srivastava A, Kumar S, Mahajan S (2021). Evaluating the effect of strengthening nurse midwifery pre-service education in two Indian states: a single group pre- and post-intervention study. Nurse Educ Today.

[25] Neil-Sztramko SE, Coletta G, Dobbins M, Marr S (2020). Impact of the AGE-ON tablet training program on social isolation, loneliness, and attitudes toward technology in older adults: single-group pre-post study. JMIR Aging.

[26] VanderKaay S, Letts L, Jung B, Moll SE (2019). On-line ethics education for occupational therapy clinician-educators: a single-group pre-/post-test study. Disabil Rehabil.

[27] Tuomikoski AM, Ruotsalainen H, Mikkonen K, Miettunen J, Kaariainen M (2018). The competence of nurse mentors in mentoring students in clinical practice—a cross-sectional study. Nurse Educ Today.

[28] Oikarainen A, Mikkonen K, Tuomikoski AM, Elo S, Pitkanen S, Ruotsalainen H (2018). Mentors’ competence in mentoring culturally and linguistically diverse nursing students during clinical placement. J Adv Nurs.

[29] Hicks C, Hennessy D (1997). The use of a customized training needs analysis tool for nurse practitioner development. J Adv Nurs.

[30] Hicks C, Hennessy D, Barwell F (1996). Development of a psychometrically valid training needs analysis instrument for use with primary health care teams. Health Serv Manag Res.

[31] Wangensteen S, Finnbakk E, Adolfsson A, Kristjansdottir G, Roodbol P, Ward H (2018). Postgraduate nurses’ self-assessment of clinical competence and need for further training. A European cross-sectional survey. Nurse Educ Today.

[32] Pinxten WJL, Fitriana E, De Jong C, Klimas J, Tobin H, Barry T (2019). Excellent reliability and validity of the addiction medicine training need assessment scale across four countries. J Subst Abuse Treat.

[33] Shewade HD, Jeyashree K, Kalaiselvi S, Palanivel C, Panigrahi KC (2016). Assessment of community-based training of medical undergraduates: development and validation of a competency-based questionnaire. Educ Health.

[34] Ellard DR, Chimwaza W, Davies D, O'Hare JP, Kamwendo F, Quenby S (2014). Can training in advanced clinical skills in obstetrics, neonatal care and leadership, of non-physician clinicians in Malawi impact on clinical services improvements (the ETATMBA project): a process evaluation. BMJ Open.

[35] Wilhelm TJ, Mothes H, Chiwewe D, Mwatibu B, Kahler G (2012). Gastrointestinal endoscopy in a low budget context: delegating EGD to non-physician clinicians in Malawi can be feasible and safe. Endoscopy.

[36] Wilson A, Lissauer D, Thangaratinam S, Khan KS, MacArthur C, Coomarasamy A (2011). A comparison of clinical officers with medical doctors on outcomes of caesarean section in the developing world: meta-analysis of controlled studies. BMJ.

[37] Murphy GT, Goma F, MacKenzie A, Bradish S, Price S, Nzala S (2014). A scoping review of training and deployment policies for human resources for health for maternal, newborn, and child health in rural Africa. Hum Resour Health.

[38] Romero D, Maldonado L, Fuentes L, Prine L (2015). Association of reproductive health training on intention to provide services after residency: the family physician resident survey. Fam Med.

[39] Block A, Dehlendorf C, Biggs MA, McNeil S, Goodman S (2017). Postgraduate experiences with an advanced reproductive health and abortion training and leadership program. Fam Med.

[40] Sibande C (2013). “There is a lot of goodwill from the country's leadership and many people to address reproductive health issues including safe motherhood…” Interview by Thengo Kavinya. Malawi Med J.

[41] Hackworth J, Steel S, Cooksey E, DePalma M, Kahn JA (2018). Faculty members’ self-awareness, leadership confidence, and leadership skills improve after an evidence-based leadership training program. J Pediatr.

[42] Shanafelt TD, Noseworthy JH (2017). Executive leadership and physician well-being: nine organizational strategies to promote engagement and reduce burnout. Mayo Clin Proc.

[43] Negandhi P, Negandhi H, Tiwari R, Sharma K, Zodpey SP, Quazi Z (2015). Building interdisciplinary leadership skills among health practitioners in the twenty-first century: an innovative training model. Front Public Health.

[44] Alidina S, Tibyehabwa L, Alreja SS, Barash D, Bien-Aime D, Cainer M (2021). A multimodal mentorship intervention to improve surgical quality in Tanzania’s Lake Zone: a convergent, mixed methods assessment. Hum Resour Health.

[45] Manzi A, Hirschhorn LR, Sherr K, Chirwa C, Baynes C, Awoonor-Williams JK (2017). Mentorship and coaching to support strengthening healthcare systems: lessons learned across the five population health implementation and training partnership projects in sub-Saharan Africa. BMC Health Serv Res.

[46] Matus J, Walker A, Mickan S (2018). Research capacity building frameworks for allied health professionals—a systematic review. BMC Health Serv Res.

[47] Van der Veken K, Belaid L, Delvaux T, De Brouwere V (2017). Research capacity building through North-South-South networking: towards true partnership? An exploratory study of a network for scientific support in the field of sexual and reproductive health. Health Res Policy Syst.

[48] Kabra R, Castillo M, Melian M, Ali M, Say L, Gulmezoglu AM (2017). Research capacity strengthening for sexual and reproductive health: a case study from Latin America. Reprod Health.

[49] Creanga AA, Jiwani S, Das A, Mahapatra T, Sonthalia S, Gore A (2020). Using a mobile nurse mentoring and training program to address a health workforce capacity crisis in Bihar, India: impact on essential intrapartum and newborn care practices. J Glob Health.

